# Postoperative Glaucoma Decompensation Following Spine Surgery: The Importance of Proper Patient Positioning

**DOI:** 10.7759/cureus.73603

**Published:** 2024-11-13

**Authors:** Tshiunza M Cherubin, Ntalaja Jeff, Mirenge Goert, Metre Guelord, Israël Maoneo, Pierre Mukuetala, Michel Kisubi, Gervith Reyes Soto, Nicola Montemurro, Manuel de Jesus Encarnacion Ramirez

**Affiliations:** 1 Department of Neurosurgery, Clinique Ngaliema, Kinshasa, COD; 2 Department of Neurosurgery, Centre Hospitalier Initiative Plus, Kinshasa, COD; 3 Department of Neurosurgery, Centre Hj Hospitals, Kinshasa, COD; 4 Department of Neurosurgery, Université de Kinshasa, Kinshasa, COD; 5 Department of Neurosurgical Oncology, Mexico National Cancer Institute, Tlalpan, MEX; 6 Department of Neurosurgery, Azienda Ospedaliero Universitaria Pisana (AOUP), Pisa, ITA; 7 Department of Neurosurgery, Instituto Nacional de Cancerología (INCAN), Mexico City, MEX; 8 Department of Digital Anatomy, United Nations Educational, Scientific and Cultural Organization (UNESCO), Paris, FRA; 9 Department of Neurological Surgery, Peoples Friendship University of Russia, Moscow, RUS

**Keywords:** patient monitor positioning, postoperative glaucoma, spine surgery, surgical complication, transforaminal lumbar interbody fusion (tlif)

## Abstract

Transforaminal lumbar interbody fusion (TLIF) is a widely utilized surgical procedure for the treatment of degenerative lumbar spinal conditions, including lumbar disc herniations, spinal stenosis, and spondylolisthesis. One such rare and underreported complication is vision loss following spinal surgery. Postoperative vision loss (POVL) is an extremely uncommon complication, occurring in approximately 0.002% to 0.2% of all non-ocular surgeries, including spinal procedures.

We presented a 70-year-old male with complaints of left-sided cruralgia, lumbar radicular pain (sciatica-type L5), and right-sided weakness who underwent L4-S1 TLIF and who reported complete vision loss in his left eye, accompanied by persistent tearing from the affected eye whenever he attempted to focus his vision. The patient’s vision in the left eye, which had been stable and functional prior to surgery, was permanently affected by the intraoperative complication. At the three-month follow-up, ophthalmological assessments confirmed that the optic nerve damage was irreversible, and the patient's vision in the left eye remained completely blurred.

Postoperative vision loss (POVL) is a rare but devastating complication associated with various types of surgeries, including spinal procedures like transforaminal lumbar interbody fusion (TLIF). The present case of a 70-year-old patient developing permanent vision loss in his left eye due to glaucoma decompensation after TLIF underscores the importance of proper intraoperative positioning, especially in patients with pre-existing ocular conditions.

## Introduction

Transforaminal lumbar interbody fusion (TLIF) is a widely utilized surgical procedure for the treatment of degenerative lumbar spinal conditions, including lumbar disc herniations, spinal stenosis, and spondylolisthesis. By providing direct access to the intervertebral disc space, TLIF allows surgeons to achieve spinal stabilization and decompression while preserving posterior spinal structures and avoiding excessive retraction of the spinal cord [1]. The approach has gained popularity due to its efficacy in restoring spinal alignment, relieving nerve compression, and achieving solid fusion across the affected vertebral levels [2]. Despite its well-established success in treating various lumbar spinal pathologies, TLIF is not without complications. Some of the most commonly reported complications include dural tears, nerve root injuries, infection, hardware failure, and non-union [3,4]. While the risk of neurological damage and implant failure is generally low, these complications can lead to significant morbidity and may require revision surgery [5]. However, rare complications that are less frequently encountered can pose unique challenges to surgeons and may not be well-represented in the existing literature.

One such rare and underreported complication is vision loss following spinal surgery. Postoperative vision loss (POVL) is an extremely uncommon complication, occurring in approximately 0.002% to 0.2% of all non-ocular surgeries, including spinal procedures [6]. Although rare, POVL can have devastating consequences for the patient, ranging from partial to complete loss of vision, and its occurrence is associated with a range of potential causes, such as increased intraocular pressure (IOP), optic nerve ischemia, and external ocular compression during surgery [7]. Among spinal surgeries, the prone positioning required for TLIF poses a specific risk for external ocular compression, which may lead to ocular complications, including glaucoma decompensation, retinal ischemia, or central retinal artery occlusion [8].

Glaucoma is a progressive optic neuropathy characterized by damage to the optic nerve, typically associated with elevated intraocular pressure. In patients with pre-existing glaucoma, even small increases in intraocular pressure or minor trauma to the eye during surgery can trigger rapid decompensation, leading to significant and potentially irreversible vision loss [9]. In this context, meticulous attention to head positioning and ocular protection during spinal surgeries, such as TLIF, becomes paramount to prevent complications related to elevated IOP or mechanical compression [10].

In this article, we present a rare case of postoperative vision loss in a 70-year-old patient following a TLIF procedure for degenerative lumbar spine disease. The patient developed complete vision loss in the left eye due to decompensation of pre-existing glaucoma, which was attributed to improper head positioning during the surgery. This case underscores the importance of a thorough preoperative assessment of ocular health in patients with known risk factors, such as glaucoma, and highlights the critical role of intraoperative positioning in preventing ocular complications.

POVL is an uncommon but well-documented complication in various surgical disciplines, with spinal surgeries posing particular risks due to the prone positioning required for adequate surgical access [11]. While ischemic optic neuropathy (ION) is primarily associated with systemic factors such as prolonged hypotension, blood loss, and anemia, external compression of the eyes during prone positioning can lead to retinal artery occlusion or exacerbate pre-existing conditions like glaucoma, as seen in our case [12,13].

The prone position, often employed during spinal surgeries, poses unique challenges in maintaining proper head and eye positioning. Ocular compression can occur if the patient’s head is improperly supported, leading to elevated intraocular pressure and potential optic nerve damage. Special headrests and positioning devices are typically used to prevent this complication, but even minor deviations in positioning can result in significant consequences for the patient [14]. In patients with pre-existing ocular conditions, such as glaucoma, the risk of decompensation during surgery is even greater, as their eyes may already be vulnerable to pressure changes and mechanical stress [8-10].

Glaucoma affects approximately 70 million people worldwide and is the leading cause of irreversible blindness [8]. The disease is characterized by progressive optic nerve damage, which is often associated with increased intraocular pressure. In patients with glaucoma, even minor elevations in IOP or episodes of optic nerve ischemia can result in rapid disease progression and irreversible vision loss. During surgery, particularly in the prone position, any pressure exerted on the eyes or head can increase IOP and place the optic nerve at further risk [9].

Our case report highlights the importance of careful intraoperative management for patients with glaucoma undergoing spinal surgery. The 70-year-old patient had a known history of glaucoma in the left eye, which had been medically managed with eye drops prior to the surgery. However, due to improper head positioning during the TLIF procedure, the patient experienced external compression of both eyes, leading to a rapid decompensation of glaucoma in the left eye. Postoperatively, the patient suffered from complete vision loss in the affected eye, and despite subsequent glaucoma surgery, the vision loss persisted.

## Case presentation

**Figure 1:**
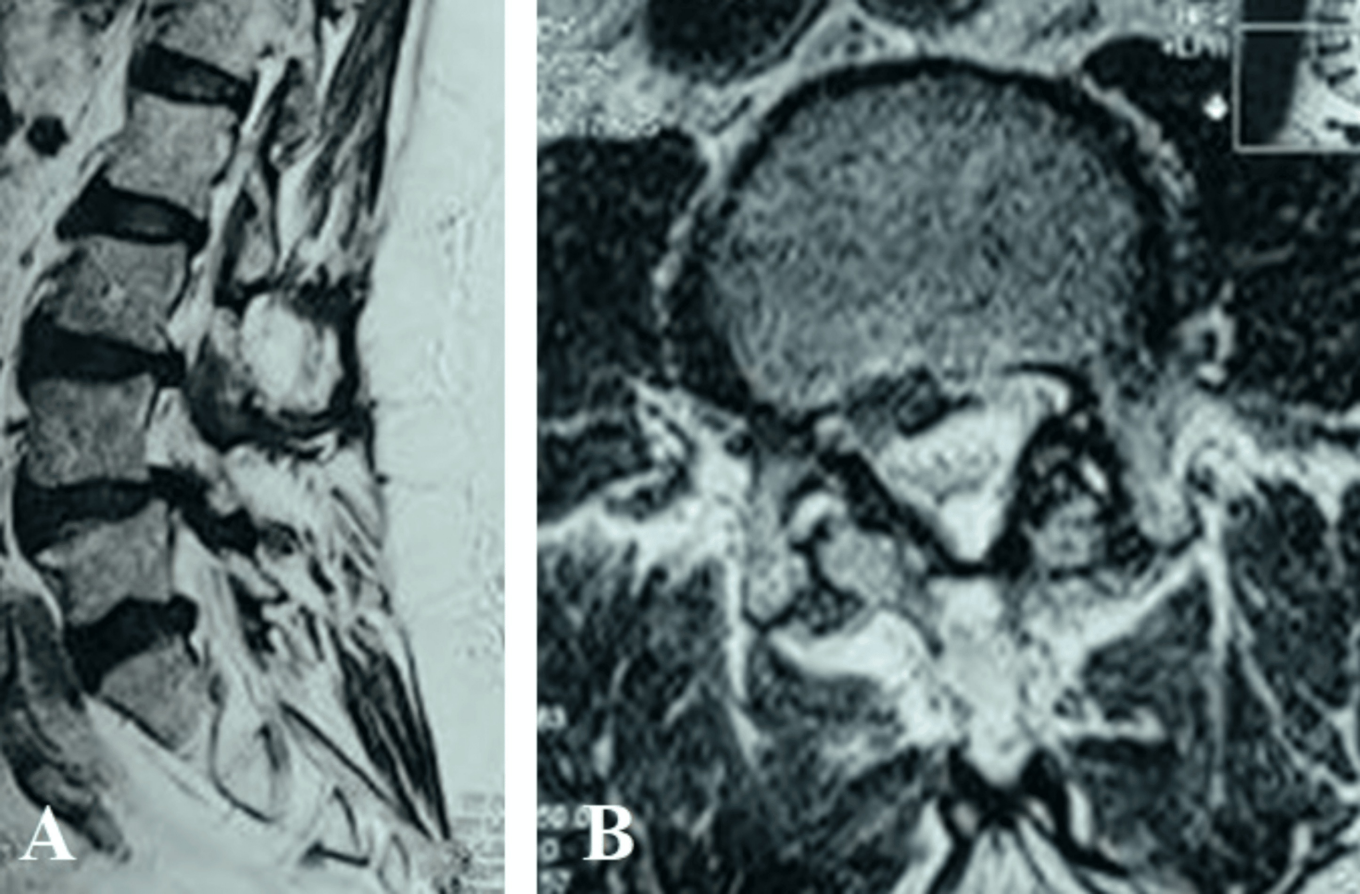
Preoperative sagittal (A) and axial (B) magnetic resonance of the lumbar spine

We presented a 70-year-old male with complaints of left-sided cruralgia, lumbar radicular pain (sciatica-type L5), and right-sided weakness. He reported paresthesias in both lower limbs, decreased walking distance, and occasional urinary incontinence. His medical history included thrombophlebitis of the lower limbs in 2009 and glaucoma in the left eye, diagnosed in 2017. The patient's preoperative clinical examination revealed a strength deficit of 4/5 in the right L5 dermatome, and both Lasègue and Wassermann signs were positive bilaterally, indicating significant nerve root compression. The patient's general condition was stable, and his vital signs were within normal ranges prior to surgery. A lumbar MRI was performed, which revealed protrusive posterior median disc herniations at the L3-L4 and L4-L5 levels, causing significant compression of the nerve roots and the dural sac. In addition, there was substantial disc space narrowing and inflammation of the vertebral endplates from L3 to S1. Based on these imaging findings, the patient was diagnosed with lumbar spinal stenosis and degenerative disc disease. The decision was made to proceed with a TLIF procedure to decompress the nerve roots and stabilize the affected vertebral segments (Figure 1).

Intraoperative course

The surgery was performed under general anesthesia, with the patient positioned prone on the operating table to allow optimal access to the lumbar spine at HJ Hospitals, Limete, Kinshasa. Standard TLIF techniques were employed, including the placement of interbody cages and pedicle screws at the L3-L4 and L4-L5 levels. Intraoperative neuromonitoring was used to ensure the preservation of nerve function throughout the procedure. However, a critical issue with head positioning occurred during the surgery. The custom-made round headrest, designed to support the patient's face and protect the eyes from compression, was improperly positioned. This resulted in prolonged bilateral ocular compression, particularly affecting the patient's left eye, which had a pre-existing glaucoma diagnosis. Although the surgery itself proceeded without complications and the desired lumbar stabilization was achieved, the intraoperative head positioning error was not recognized until the patient awoke in the post-anesthesia care unit.

Postoperative course and ophthalmological evaluation

Upon awakening, the patient reported complete vision loss in his left eye, accompanied by persistent tearing from the affected eye whenever he attempted to focus his vision. Ophthalmological consultation was urgently requested, and a comprehensive examination revealed that the patient's glaucoma had decompensated, likely due to the prolonged external compression of the eye during surgery. IOP was significantly elevated in the left eye, and optic nerve damage was suspected. Initial medical management included the use of glaucoma eye drops to reduce intraocular pressure and prevent further damage to the optic nerve. However, after three weeks of observation, there was no improvement in the patient's vision, prompting the decision to perform glaucoma surgery. Unfortunately, despite this intervention, the patient's vision remained severely compromised, with complete blurring of vision in the left eye persisting at the three-month follow-up.

Visual outcomes

The patient's vision in the left eye, which had been stable and functional prior to surgery, was permanently affected by the intraoperative complication. At the three-month follow-up, ophthalmological assessments confirmed that the optic nerve damage was irreversible, and the patient's vision in the left eye remained completely blurred. This complication significantly impacted the patient's postoperative recovery and overall quality of life, as the vision loss in one eye affected his daily activities and independence.

In contrast, the patient's right eye, which was also exposed to some degree of intraoperative compression, did not exhibit significant changes in vision. This outcome highlights the particular vulnerability of the left eye, which had a pre-existing diagnosis of glaucoma, making it more susceptible to external pressure and elevated intraocular pressure during surgery.

Spinal surgery outcomes

**Figure 2:**
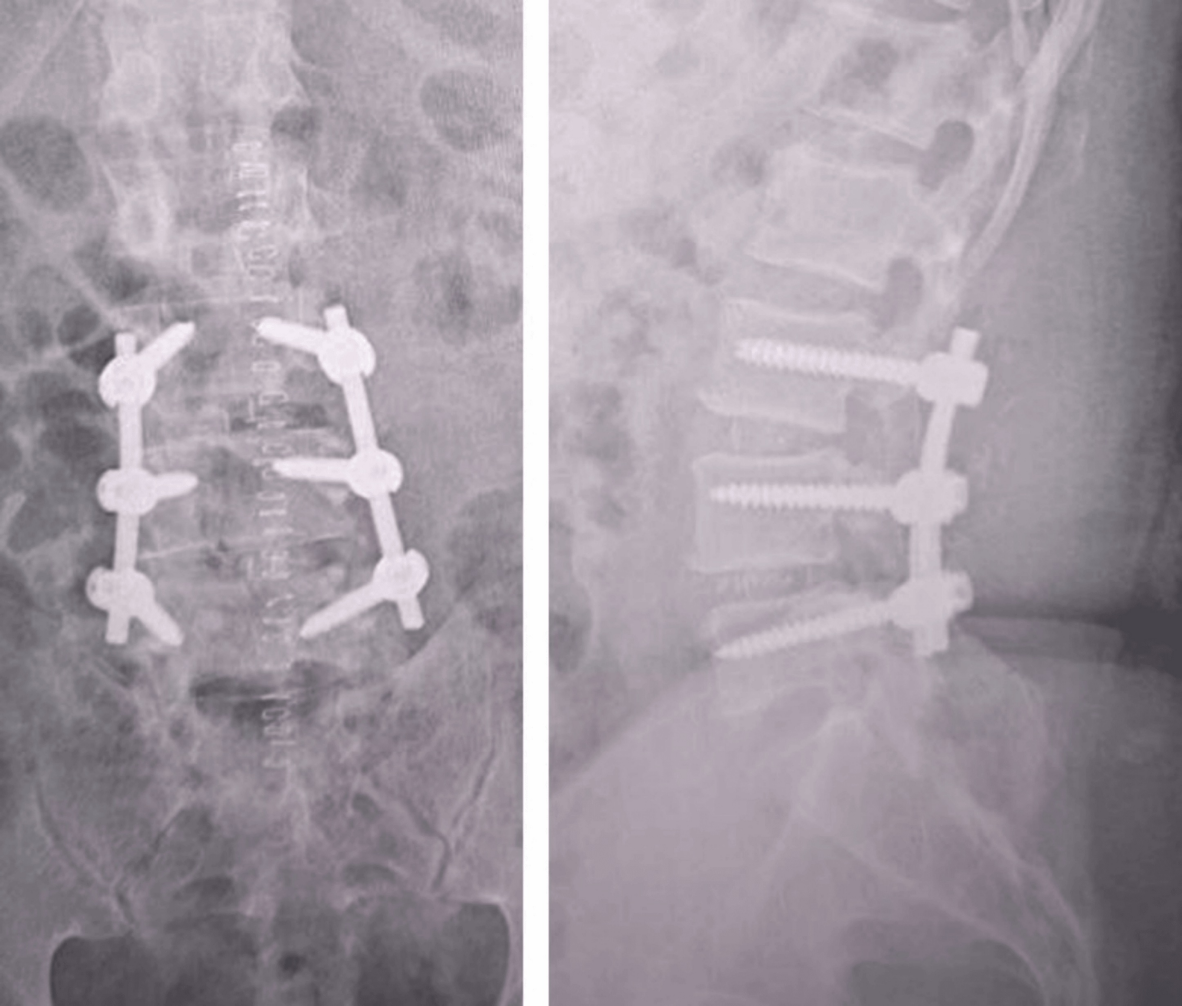
Postoperative X-ray of the lumbar spine

Despite the severe ophthalmological complication, the patient's spinal surgery achieved its intended goals. The TLIF procedure successfully decompressed the nerve roots at L3-L4 and L4-L5, alleviating the patient's lumbar radicular symptoms. At the three-month postoperative follow-up, the patient reported significant improvement in pain and mobility, with no residual neurological deficits in the lower limbs. The surgical site healed well, and there were no signs of infection or hardware failure. The patient was able to gradually resume normal activities with physical rehabilitation, although the visual impairment continued to limit his full recovery (Figure 2).

Preventive measures and institutional changes

In response to this case, the surgical team at HJ Hospitals implemented several critical changes to prevent similar complications in future spinal surgeries. Silicone headrests were introduced to replace the custom-made round headrests, which had been identified as a contributing factor to ocular compression. The silicone headrests were designed to evenly distribute pressure around the patient's head and face, reducing the risk of eye compression during prone positioning. Additionally, the institution's surgical checklist was revised to include specific measures for ensuring proper head and eye positioning. This checklist now includes verification that the eyes are completely free from contact with the headrest, as well as additional steps to ensure that other vulnerable areas, such as the abdomen and ankles, are properly positioned and protected. These preventive measures aim to reduce the likelihood of postoperative vision loss and other positioning-related complications in future cases.

## Discussion

POVL is a rare but devastating complication associated with various types of surgeries, including spinal procedures like TLIF. The present case of a 70-year-old patient developing permanent vision loss in his left eye due to glaucoma decompensation after TLIF underscores the importance of proper intraoperative positioning, especially in patients with pre-existing ocular conditions. This discussion will explore the risk factors, pathophysiology, and prevention strategies associated with POVL in spine surgeries, with a particular focus on prone positioning, glaucoma management, and institutional improvements for mitigating such complications.

POVL following non-ocular surgeries, particularly in spinal surgeries, can occur due to several underlying mechanisms. These include ischemic optic neuropathy (ION), central retinal artery occlusion (CRAO), cortical blindness, and external compression of the globe leading to increased IOP [13]. The prone positioning used during most spinal surgeries, including TLIF, presents unique challenges in preventing these complications. The two most common forms of ION are anterior ischemic optic neuropathy (AION) and posterior ischemic optic neuropathy (PION), both of which are characterized by infarction of the optic nerve head or the posterior portion of the optic nerve, respectively [14].

While ION typically arises from systemic factors such as prolonged hypotension, anemia, or significant blood loss during surgery, external compression of the eye can lead to CRAO or glaucomatous optic neuropathy [15]. In our case, the patient's pre-existing glaucoma likely predisposed him to the severe ocular consequences of improper positioning. Elevated IOP, either from prone positioning or direct ocular compression, could exacerbate optic nerve damage in glaucoma patients, leading to rapid decompensation and irreversible vision loss [16].

According to Lee et al., external ocular compression in prone-positioned patients has been linked to increased IOP, especially when head support fails to adequately relieve pressure on the eyes [17]. Our case highlights this issue, as improper positioning of the headrest resulted in bilateral ocular compression, with the left eye already compromised by glaucoma-experiencing decompensation. While most cases of POVL are multifactorial, it is important to recognize that pre-existing ocular conditions such as glaucoma significantly increase the risk of vision loss, even in the absence of other systemic complications [18].

Glaucoma, a progressive optic neuropathy, is the leading cause of irreversible blindness worldwide. It is primarily characterized by optic nerve damage and visual field loss due to increased IOP. Patients with glaucoma are particularly vulnerable during surgeries that require prone positioning, such as TLIF, where external ocular compression can dramatically elevate IOP, leading to rapid disease progression [19]. In our case, the patient had a history of glaucoma in the left eye, which had been managed with topical medications. However, intraoperative ocular compression during TLIF likely caused a sudden spike in IOP, overwhelming the optic nerve's capacity to cope and resulting in permanent vision loss [20].

Li et al. have shown that elevated IOP during prone surgery is a significant risk factor for optic neuropathy in patients with glaucoma [21]. This is particularly concerning in prolonged surgeries or procedures that require significant intraoperative hypotension. In cases where the head is inadequately supported or where there is direct ocular pressure, glaucoma patients are at an even greater risk of decompensation. This makes preoperative screening and intraoperative precautions critical for preventing such outcomes [22].

The key to preventing complications like POVL in spinal surgeries lies in meticulous patient positioning and real-time intraoperative monitoring of ocular pressure and perfusion. The prone position is necessary for adequate exposure of the lumbar spine during TLIF, but it comes with inherent risks, including external ocular compression and reduced venous outflow from the head, both of which can contribute to elevated IOP [23]. Various techniques have been proposed to mitigate these risks, including the use of specialized headrests, eye protection devices, and intraoperative IOP monitoring.

In this case, the improper use of a custom-made headrest led to bilateral ocular compression, contributing to the patient's postoperative vision loss. As highlighted by Warner et al., prone-positioned patients should have their heads supported by silicone or gel-based headrests designed to relieve pressure from the eyes, ensuring that the globes are not compressed and that venous drainage is optimized [24]. Postoperatively, ocular complications like glaucoma decompensation may not always be immediately apparent, and close monitoring of visual function is crucial, especially in high-risk patients like those with pre-existing glaucoma [25].

Recent advancements in intraoperative monitoring have also introduced the potential for real-time assessment of IOP and optic nerve perfusion during surgery, though these techniques are not yet widely adopted in routine practice [26]. Future directions in preventing POVL may involve the integration of IOP monitoring systems into operating rooms, allowing surgeons to identify dangerous pressure elevations before they result in permanent damage [27]. In our case, the failure to detect and prevent ocular compression underscores the need for more robust monitoring systems to protect at-risk patients.

Preoperative screening for ocular conditions, particularly in older patients or those with known risk factors such as glaucoma, is essential in preventing postoperative visual complications. Patients undergoing spinal surgeries, especially in the prone position, should undergo comprehensive ophthalmologic evaluations to identify any pre-existing conditions that could increase their risk for POVL [28]. In our case, the patient had a known diagnosis of glaucoma, yet the risks associated with intraoperative IOP elevation were not adequately mitigated.

Adequate informed consent is also crucial in such cases. Surgeons must inform patients about the potential risk of vision loss, particularly in individuals with pre-existing ocular conditions, and discuss the steps that will be taken to minimize these risks [29]. As recommended by Shir Yen et al. and Moss et al., preoperative discussions should include details on the potential for visual complications and the measures employed to protect the patient's eyes during surgery [30,31]. In cases where significant risk factors are identified, patients should be made aware of the possibility of POVL, even if the risk is exceedingly low.

Postoperative management of POVL

The management of POVL requires timely diagnosis and intervention to prevent further deterioration of vision. In the immediate postoperative period, any patient reporting visual changes should undergo an urgent ophthalmologic evaluation. In our case, the patient developed complete vision loss in the left eye immediately after the TLIF procedure, and an ophthalmology consultation was sought. Despite initial attempts to manage the condition medically with topical glaucoma medications, there was no significant improvement, and the patient ultimately required glaucoma surgery [31].

Unfortunately, despite surgical intervention, the patient's vision remained permanently compromised. This outcome underscores the difficulty in reversing optic nerve damage once it occurs, particularly in patients with pre-existing conditions like glaucoma. According to Furlan et al., early intervention is critical, but once significant optic nerve damage has occurred, the prognosis for visual recovery is often poor [32]. In this case, the optic nerve damage was likely too advanced by the time treatment was initiated, and the patient's visual function could not be restored.

Institutional and systemic improvements

This case has prompted significant institutional changes at HJ Hospitals to prevent similar complications in future patients. The surgical team has adopted new positioning protocols, including the use of silicone-based headrests designed to distribute pressure evenly and avoid ocular compression. In addition, the hospital's surgical checklists have been revised to include specific steps for verifying the patient's head and eye positioning before and during the procedure [33]. These checklists now include ensuring that the eyes are free from any pressure, the abdomen is properly supported, and the lower extremities are correctly aligned to prevent excessive strain or compression.

The adoption of these new protocols aligns with recommendations from major surgical societies, which advocate for the use of standardized positioning protocols and checklists to reduce the incidence of intraoperative complications [34-38]. Studies by Moss et al. have shown that the implementation of such protocols can significantly reduce the incidence of positioning-related complications in spinal surgeries, including POVL [35,39-40]. By improving institutional practices and promoting a culture of safety, hospitals can better protect patients from preventable complications.

Key lessons and implications

Preoperative Screening for Ocular Conditions

This case demonstrates the importance of comprehensive preoperative screening for patients with known ocular conditions such as glaucoma. A detailed ophthalmologic assessment, including IOP measurements and optic nerve evaluations, should be part of the routine preoperative workup for patients undergoing prone-positioned surgeries. Identifying patients at increased risk for ocular complications allows for more targeted interventions, such as modified positioning strategies or intraoperative monitoring of IOP, to reduce the likelihood of vision loss.

Intraoperative Positioning and Head Support

Proper intraoperative head positioning is paramount in preventing complications such as POVL. In this case, the use of a custom-made headrest led to inadvertent ocular compression, resulting in glaucoma decompensation. The introduction of silicone-based headrests, which more evenly distribute pressure and prevent direct ocular compression, offers a simple but effective solution to this problem. Incorporating standardized positioning protocols and verifying the positioning of the head and eyes throughout the procedure should be part of every surgical team's practice.

Intraoperative Monitoring

While real-time IOP monitoring during spinal surgeries is not yet common practice, it may become increasingly relevant, particularly for high-risk patients. The use of intraoperative monitoring tools to assess IOP and optic nerve perfusion could provide critical data that would allow surgeons to intervene before permanent damage occurs. As technology advances, these monitoring systems could become part of routine care, improving patient safety and outcomes in spinal surgeries.

Informed Consent and Patient Education

It is essential that patients are fully informed of all potential risks associated with surgery, including rare but severe complications like POVL. In this case, the patient had pre-existing glaucoma, a condition that heightened his risk for vision loss, particularly under conditions of elevated IOP. Surgeons must ensure that patients understand the risks associated with their specific comorbidities and the steps that will be taken to mitigate these risks during surgery. This fosters better patient engagement and ensures that they are prepared for all possible outcomes.

Multidisciplinary Approach

A key aspect of this case is the importance of multidisciplinary collaboration in the care of complex patients. Preoperative consultations between neurosurgeons, anesthesiologists, and ophthalmologists could have highlighted the patient's high risk of ocular complications due to glaucoma. A more integrated approach could lead to more personalized care, better intraoperative decision-making, and ultimately, better outcomes for patients with complex medical histories.

Institutional Changes and Surgical Checklists

As a direct result of this case, HJ Hospitals implemented institutional changes aimed at preventing future occurrences of POVL. This includes the adoption of silicone-based headrests, revised positioning protocols, and updated surgical checklists that emphasize patient safety in prone-positioned surgeries. These changes align with best practices and recommendations from surgical societies, which advocate for standardized protocols to reduce the incidence of positioning-related complications. Other institutions can learn from this case and adopt similar measures to improve patient outcomes in spinal surgeries.

## Conclusions

This case highlights the rare but serious complication of POVL following TLIF, specifically due to glaucoma decompensation triggered by improper intraoperative positioning. Although TLIF is a widely utilized and generally successful procedure for managing lumbar degenerative conditions, this case underscores the critical importance of paying meticulous attention to patient positioning, particularly in prone surgeries where ocular compression is a risk factor. While POVL is a rare complication, its potential to cause permanent and life-altering outcomes necessitates a proactive and preventive approach. This case serves as a stark reminder that even the most routine of procedures carries risks, and it is the responsibility of every surgical team to minimize those risks through careful planning, vigilance, and adherence to best practices. By learning from this case and implementing targeted preventive strategies, we can improve the safety of spinal surgeries and reduce the incidence of POVL, ensuring better outcomes for patients undergoing these complex procedures.
